# Effect of acute citalopram on self-referential emotional processing and social cognition in healthy volunteers

**DOI:** 10.1192/bjo.2020.107

**Published:** 2020-10-19

**Authors:** Catherine Hobbs, Susannah E. Murphy, Lucy Wright, James Carson, Indra Van Assche, Jessica O'Brien, Mayowa Oyesanya, Jie Sui, Marcus R. Munafò, David Kessler, Catherine J. Harmer, Katherine S. Button

**Affiliations:** Department of Psychology, University of Bath, UK; Department of Psychiatry, University of Oxford, and NHS Foundation Trust, Warneford Hospital, Oxford, UK; Department of Psychiatry, University of Oxford, and NHS Foundation Trust, Warneford Hospital, Oxford, UK; Department of Psychiatry, University of Oxford, and NHS Foundation Trust, Warneford Hospital, Oxford, UK; Department of Psychiatry, University of Oxford, and NHS Foundation Trust, Warneford Hospital, Oxford, UK; Department of Psychiatry, University of Oxford, and NHS Foundation Trust, Warneford Hospital, Oxford, UK; Department of Psychiatry, University of Oxford, and NHS Foundation Trust, Warneford Hospital, Oxford, UK; School of Psychology, University of Aberdeen, UK; School of Psychological Science, University of Bristol, MRC Integrative Epidemiology Unit at the University of Bristol, and National Institute of Health Research Biomedical Research Centre at the University Hospitals Bristol NHS Foundation Trust and the University of Bristol, UK; Population Health Sciences, University of Bristol; Department of Psychiatry, University of Oxford, and NHS Foundation Trust, Warneford Hospital, Oxford, UK; Department of Psychology, University of Bath, UK

**Keywords:** Antidepressants, cognitive neuroscience, social functioning, psychological testing, depressive disorders

## Abstract

**Background:**

Depression is characterised by negative views of the self. Antidepressant treatment may remediate negative self-schema through increasing processing of positive information about the self. Changes in affective processing during social interactions may increase expression of prosocial behaviours, improving interpersonal communications.

**Aims:**

To examine whether acute administration of citalopram is associated with an increase in positive affective learning biases about the self and prosocial behaviour.

**Method:**

Healthy volunteers (*n* = 41) were randomised to either an acute 20 mg dose of citalopram or matched placebo in a between-subjects double-blind design. Participants completed computer-based cognitive tasks designed to measure referential affective processing, social cognition and expression of prosocial behaviours.

**Results:**

Participants administered citalopram made more cooperative choices than those administered placebo in a prisoner's dilemma task (β = 20%, 95% CI: 2%, 37%). Exploratory analyses indicated that participants administered citalopram showed a positive bias when learning social evaluations about a friend (β = 4.06, 95% CI: 0.88, 7.24), but not about the self or a stranger. Similarly, exploratory analyses found evidence of increased recall of positive words and reduced recall of negative words about others (β = 2.41, 95% CI: 0.89, 3.93), but not the self, in the citalopram group.

**Conclusions:**

Participants administered citalopram showed greater prosocial behaviours, increased positive recall and increased positive learning of social evaluations towards others. The increase in positive affective bias and prosocial behaviours towards others may, at least partially, be a mechanism of antidepressant effect. However, we found no evidence that citalopram influenced self-referential processing.

Addressing negative perceptions of the self is believed to be central to the successful treatment of depression. According to cognitive theories, individuals with depression hold negative views and expectations about the self, developed as an internalised response to repeated negative social experiences (e.g. peer victimisation). When activated by external stressors, these negative self-schema dominate information processing, increasing automatic processing of negative information about the self. Deliberative cognitive processing is attenuated, preventing reappraisal of these automatic biases.^1^ Supportive of this theory, peer victimisation in childhood is associated with increased negative and reduced positive perceptions of the self.^2^ Furthermore, negative affective biases are more likely to be observed in depression if stimuli are encoded in reference to the self.^2–5^ Changes in self-referential affective processing may therefore be a key mechanism of treatments for depression.

## The role of antidepressants in addressing negative self-schema

Depression is commonly treated using antidepressant medication.^6^ Antidepressants are believed to operate by remediating negative affective biases early in treatment.^7^ Supportive of this, short-term administration of antidepressants is associated with increased processing of positive stimuli in both depressed and healthy volunteers.^8^ Negative self-schemas may be addressed through these changes in automatic affective processing.^9^ Individuals with depression preferentially process negative information about the self.^10^ Shifting affective processing through antidepressant treatment may expose individuals to more positive information about the self, remediating negative schema. In keeping with this argument, antidepressants have been found to increase recall of positive characteristics encoded to the self in healthy and depressed individuals.^11,12^

## Importance of social cognition

In order for changes in affective processing from antidepressant treatment to alter mood, it has been suggested that individuals must engage with their social environment to relearn associations in a more positive context.^13^ However, this aspect of the model is yet to be fully explored. Greater depression severity is associated with poorer-quality social interactions^14^ and increased expectations of rejection.^15^ Raised expectations of rejection may evoke hostile or non-responsive social behaviours, increasing the likelihood of reciprocal negative behaviours from others and reinforcing negative expectations of social interactions.^16,17^ In keeping with previous evidence of increases in positive affective biases and behaviours following antidepressant administration,^11,12,18^ antidepressant treatment is likely to strengthen positive learning and prosocial behaviours during social interactions. Repeated social interactions with remediated positive affective biases may therefore reinforce engagement in future social interactions, potentially addressing the issues of social withdrawal that are characteristic of depression.

## Altering self-schema through changes in social cognition

The self is a social construct, shaped by our perceptions of others’ evaluations of us.^19^ During social interactions, healthy individuals preferentially incorporate positive evaluations into their self-concept.^20^ By contrast, individuals with greater depression expect more negative evaluations,^21^ selectively engage in negative feedback^22^ and show reduced learning of positive evaluations^23^ about the self. Preferential engagement with negative social evaluations about the self may reinforce negative self-schema and increase social withdrawal in a vicious cycle. Increasing positive affective biases through antidepressant treatment may increase learning of positive social evaluations from others, altering the affective content of self-schema and breaking the pattern of maladaptive learning in social interactions. Changes in self-referential affective learning within social contexts may be an important pathway in antidepressant action.

## Aims

In this study, we examined the influence of acute administration of citalopram on affective self-referential cognition and social behaviours in healthy volunteers using a double-blind placebo-controlled design. We hypothesised that acute administration of citalopram would be associated with an increase in positive affective biases about the self and increased prosocial behaviour.

## Method

This study was pre-registered on the Open Science Framework (https://osf.io/nhjvs/), where study materials are also available. The data that support the findings of this study are openly available in the University of Bath Research Data Archive at https://doi.org/10.15125/BATH-00891.

### Subjects

Participants aged 18–45 years and fluent in English, with normal or corrected-to-normal vision, were recruited through advertisement to the local community. We excluded participants meeting diagnostic criteria for past or current axis 1 DSM-V psychiatric disorder identified using the Structured Clinical Interview for DSM-V axis I disorders (SCID-V^24^). Other exclusion criteria were current use of psychoactive medication (excluding contraceptive medication), current or past drug or alcohol dependency, a current or past significant neurological condition, known hypersensitivity to the study drug, current pregnancy or breast feeding, current significant medical condition, consumption of more than five cigarettes or more than six caffeinated drinks per day, lactose intolerance, previous participation in a study using similar cognitive tasks, previous participation in a study involving medication within the past 3 months, or recreational psychoactive drug use within the past 3 months.

### Ethical approval

The authors assert that all procedures contributing to this work comply with the ethical standards of the relevant national and institutional committees on human experimentation, and with the Helsinki Declaration of 1975, as revised in 2008. All procedures involving human subjects were approved by the University of Oxford Medical Sciences Interdivisional Research Committee (R64589). Written informed consent was obtained from all participants.

### Design

This study used a between-subject, double-blind, placebo controlled design. Participants were randomised to receive a single acute oral dose of 20 mg citalopram or lactose placebo encapsulated in identical white capsules. Blocked randomisation, stratified by gender, was generated using an online randomisation tool.^25^

### Procedure

Participants first completed self-report questionnaires on mood and personality (baseline) and were administered the study medication. Citalopram is rapidly absorbed, with peak concentrations reached within 2–4 h.^26^ Cognitive testing therefore started following a 3 h rest period after drug administration, in order to maximise drug levels during testing. Participants repeated state measures of mood (post-drug timepoint) and completed the following cognitive tests in a fixed order: social evaluation learning; associative learning; prisoner's dilemma; go/no-go self-esteem; referential categorisation and recall. Participants then repeated the state measures of mood (post-testing timepoint), before completing the Oxford Cognition Stress Task (reported elsewhere). Testing lasted approximately 1.5–2 h. Prior to participating, participants were asked to eat a light meal and were provided with light refreshments after the rest period. Participants were informed that the study aimed to examine how citalopram alters processing of emotional and social information about the self and others, but they were blinded to the specific study hypotheses.

### Materials

#### Questionnaires

Depression was measured using the Patient Health Questionnaire (PHQ-9)^27^ and Beck Depression Inventory (BDI-II).^28^ Anxiety was measured using the Generalised Anxiety Disorder Scale (GAD-7),^29^ the Brief Fear of Negative Evaluation Scale^30^ and the Trait Anxiety Inventory.^31^ Personality traits were measured using the Eysenck Personality Questionnaire Abbreviated.^32^ State mood was measured using the State Anxiety Inventory,^31^ Positive and Negative Affect Scale (PANAS),^33^ and visual analogue scales (VAS) of sadness, disgust, anger, fear, anxiety and alertness. State mood measures were completed at baseline, post-drug and post-testing timepoints.

#### Blinding

To assess the effectiveness of blinding, at the end of testing participants and the administrating researcher guessed the study drug administered, and indicated their certainty regarding this guess using a VAS. Side-effects were also monitored using participant self-reports of nausea, dizziness, dry mouth, headaches, alertness and agitation (absent to severe) at each timepoint.

#### Cognitive tasks

For brevity, a short description of each task is provided below; full details can be found in the supplementary material, available at https://doi.org/10.1192/bjo.2020.107.

**Fig. 1:**
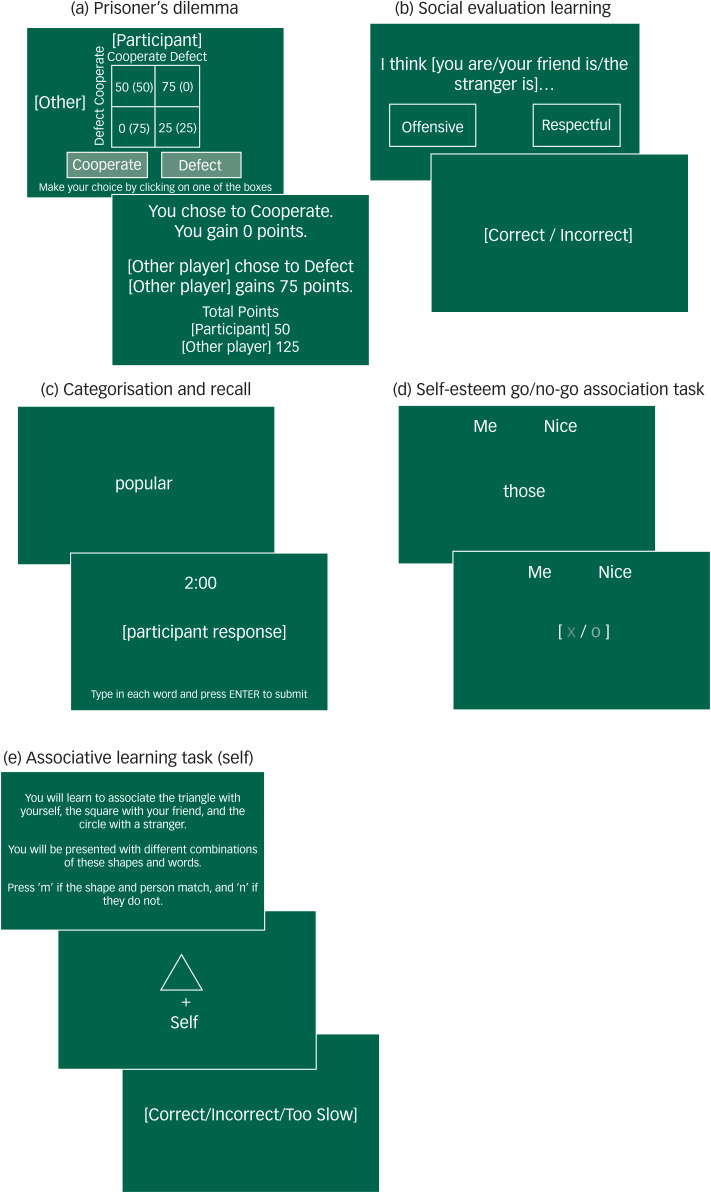
Cognitive task procedures.

Prisoners’ dilemma: Antidepressants may promote positive social relationships with others by increasing prosocial behaviours. We therefore measured cooperative behaviours using a prisoners’ dilemma task. Participants won points based on their decision to cooperate or defect in combination with the computer-simulated opponent's decision (Fig. 1(a)). If both chose to cooperate then the points were equally shared, if one defected and the other cooperated then the defector gained all the points, and if both defected neither player gained any points. Participants were unaware of the other player's decision when making their choice. Social context was manipulated so that the other player could initially choose to cooperate (positive) or defect (negative). The proportion of cooperative choices was recorded.

**Fig. 2:**
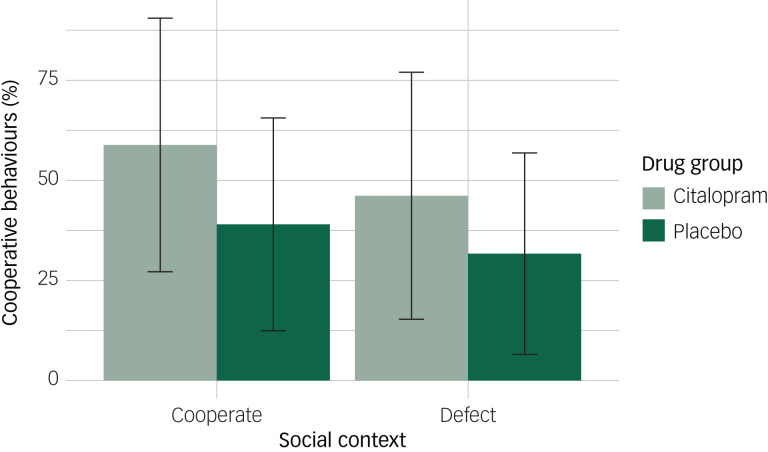
Mean proportion of cooperative behaviours according to drug group and social context. Error bars represent standard deviations.

Social evaluation learning: It is possible that antidepressants may alter negative self-schema by increasing learning of positive evaluations about the self. We therefore measured learning of social evaluations within a reinforcement learning task.^34,35^ Participants learned how much the computer ‘liked’ the self, a friend and a stranger based on feedback to a forced choice selection between positive and negative social evaluation pairings (Fig. 1(b)). Participants learned two rules based on the probability of the positive evaluations being ‘correct’ (‘like’ 60–80%, ‘dislike’ 20–40%). The number of errors made before reaching the criterion of eight consecutive rule-congruent responses was recorded. Bias scores were calculated by subtracting errors to criterion made when learning the dislike rule from the like rule.

Referential categorisation and recall: Previous research has indicated increases in recall of positive characteristics following antidepressant administration. However, the specificity of these effects to the self are unclear. To measure recall of characteristics about the self and others, participants first categorised whether 40 presented positive and negative traits described themselves or a familiar other (yes/no). Participants were then given 2 min to recall those personality traits, using the keyboard to enter their responses (Fig. 1(c)). Separate blocks were completed for each referential condition in a randomised order. The total numbers of words categorised and correctly recalled were recorded.

Self-esteem go/no-go: To measure self-referential processing occurring in interaction with affective processing we used a go/no-go task. This task measured inhibitory control when responding to affective words in relation to the self and others.^36^ Participants categorised words relating to the self or others, and positive or negative traits, by pressing the space bar if a presented word belonged to a specified paired referential-emotion category (Fig. 1(d)). Discriminative accuracy (d′) was calculated according to the referential-emotion condition.

Associative learning: To measure self, emotion and reward processing occurring independently, we used three simple associative learning tasks.^37,38^ In each task, participants were presented with a combination of stimulus-shape pairings, related to the relevant area of processing, and used the keyboard to indicate whether the presented pairings matched previously learnt associations (Fig. 1(e)). Stimuli varied according to the area of processing examined. Accuracy (percentage correct) and reaction times were recorded.

### Statistical analyses

Analyses were conducted in R 3.6.

#### Sample size calculation

We aimed to recruit 44 participants to provide 90% power to detect changes in emotion processing previously observed in healthy volunteer studies (drug mean 10.64 (s.d. 9.77), placebo mean 3.36 (s.d. 5.96)).^12^ However, owing to COVID-19, recruitment was terminated at 41 participants in March 2020. With the recruited sample, we were able to detect an effect size of d = 1.04 at 90% power and an alpha of 0.05.

#### State mood and side-effects

The influence of citalopram on state mood and side-effects were tested using mixed-effects linear regression models. Separate models were conducted for each measure, with drug group, timepoint and the interaction between these as predictors. Participant was entered as a random effect to account for the effect of time.

#### Cognitive tasks

A series of mixed-effect linear regression models were used to assess the influence of citalopram on task performance. For all models, participant was entered as a random effect to account for the repeated measures elements of tasks, drug group was entered as a predictor, and the task outcome as the outcome. For tasks including a referential and/or valence (e.g. emotion or rule) condition, these were entered into the models as additional categorical predictors, independently and in interaction with drug group. Full model details are available in the supplementary material. For exploratory analyses, *P*-values are not reported owing to undetermined inflation of the alpha rate.^39^

#### Drug group guess and certainty

Differences in group assignment guesses according to drug group were assessed using χ^2^-tests. Differences in certainty of group assignment according to drug group were evaluated using *t*-tests.

## Results

### Sample

Participants (*n* = 41) were randomly allocated to the citalopram (*n* = 20) or placebo group (*n* = 21). Sample characteristics, according to drug group, are presented in Table 1.

**Table 1:** Sample demographic characteristics and baseline trait mood and personality self-report measures

	Citalopram (*N* = 20)^a^	Placebo (*N* = 21)
Age, mean (s.d.)	23.90 (3.24)	22.86 (3.58)
Female, *N* (%)	16 (80)	17 (81)
Ethnicity, *N* (%)		
Asian	5 (25)	6 (29)
Black	0 (0)	1 (5)
Caucasian	14 (70)	12 (57)
Mixed	1 (5)	2 (9)
Occupation, *N* (%)		
Employed	5 (25)	4 (19)
Student	15 (75)	17 (81)
Educational attainment, *N* (%)		
Sixth-form college	5 (25)	9 (43)
Undergraduate	7 (33)	7 (33)
Postgraduate	8 (38)	5 (24)
English spoken as first language, *N* (%)	10 (50)	19 (90)
PHQ-9, mean (s.d.)	1.05 (1.43)	1.33 (1.93)
BDI-II, mean (s.d.)	1.58 (2.09)	2.24 (3.65)
GAD-7, mean (s.d.)	0.47 (0.70)	0.71 (1.38)
BFNE, mean (s.d.)	26.89 (7.42)	29.57 (6.61)
STAI-T, mean (s.d.)	31.16 (6.32)	32.0 (7.30)
EPQR-A, mean (s.d.)		
Extraversion	9.21 (2.42)	9.43 (3.06)
Neuroticism	2.63 (2.50)	3.00 (2.53)
Lie	4.42 (1.89)	4.81 (2.54)
Psychoticism	2.37 (1.83)	2.71 (1.93)

PHQ-9, Patient Health Questionnaire; BDI-II, Beck Depression Inventory II; GAD-7, Generalised Anxiety Disorder Questionnaire; BFNE, Brief Fear of Negative Evaluation Scale; STAI-T, State-Trait Anxiety Inventory Trait Subscale; EPQR-A, Eysenck Personality Questionnaire Abbreviated.

a Data were missing for one participant for mood and personality measures in the citalopram group (*N* = 19) owing to a technical error.

### Self-reported state mood

We found no evidence that citalopram altered mood. Participants showed some evidence of a decrease in positive mood (PANAS positive) between baseline and post-drug (β = −1.90, 95% CI: −3.77, −0.02, *P* = 0.051), and post-testing (β = −2.47, 95% CI: −4.35, −0.60, *P* = 0.012), but this did not differ between groups (post-drug: β = −0.25, 95% CI: −2.84, 2.34, *P* = 0.852, post-testing: β = −1.00, 95% CI: −3.59, 1.59, *P* = 0.450). Participants showed a slight increase in state anxiety (State-Trait Anxiety Inventory State Subscale; STAI-S) between baseline and post-testing (β = 2.31, 95% CI: 0.00, 4.63, *P* = 0.054), but this did not differ by drug group (β = 0.64, 95% CI: −2.56, 3.83, *P* = 0.697). No significant changes over time or differences between groups were observed for the PANAS negative subscale or for ratings of disgust, anger, fear, anxiety or alertness.

Differences were observed between drug groups at baseline in VAS ratings of happiness and sadness, with the placebo group showing higher levels of sadness (β = 8.86, 95% CI: 1.50, 16.22, *P* = 0.021) and lower levels of happiness (β = −8.22, 95% CI: −15.93, −0.51, *P* = 0.041). However, exploratory follow-up pairwise comparisons of drug group according to timepoint found no group differences at future timepoints (Supplementary Table 1).

### Cognitive tasks

#### Prisoner's dilemma

An effect of the drug was observed on the proportion of cooperative choices; participants in the placebo group made 20% fewer cooperative choices compared with the citalopram group (95% CI: −37%, −2%, *P* = 0.030). There was some suggestion of a main effect of social context in the expected direction, although the confidence interval included the null; on average, participants made 13% fewer cooperative choices when the other player had initially defected versus cooperated (95% CI: −26%, 1%, *P* = 0.073). We did not find evidence of an interaction between drug group and social context, indicating that participants in the placebo group made fewer cooperative choices irrespective of whether the other player initially cooperated or defected (β = 5%, 95% CI: −14%, 24%, *P* = 0.580; Figure 2).

#### Social evaluation learning

There were some indications that participants in the citalopram group showed a greater positive bias overall, as indicated by bias scores. Participants in the citalopram group, on average, made 4.8 (s.d. 6.64) more errors when learning negative relative to positive evaluations. By comparison, participants in the placebo group made 2.25 (s.d. 5.37) more errors on average when learning negative relative to positive evaluations. This group effect was particularly heightened for the friend condition (citalopram: −5.70, s.d. 5.45; placebo: −1.64, s.d. 4.59; Supplementary Fig. 1). However, in our initial confirmatory model examining main and interaction effects of the referential condition and drug group, we found no evidence of group differences (Supplementary Table 2).

To examine whether effects of bias scores were obscured by learning within a particular rule (e.g. better learning of ‘dislike’ or worse learning of ‘like’), we examined the effects of drug group on errors to criterion according to the referential condition and rule. Participants made a greater number of errors before learning the negative ‘dislike’ rule (β = 4.53, 95% CI: 2.21, 6.84, *P* < 0.001). However, this effect did not vary according to drug group or referential condition. No main effects of drug, referential condition, or an interaction between these were observed (Supplementary Table 2).

Given our previous findings of increased prosocial behaviours towards others in the citalopram group in the prisoners’ dilemma task, and given that the sample size may be small for testing interaction effects, and that the descriptive statistics suggested a drug group effect in the friend condition, we conducted additional exploratory analyses focusing on the effects of drug group in the friend condition. We therefore examined the effects of drug group on bias scores separately for each referential condition. Participants in the citalopram group showed a greater positive bias in the friend condition (β = 4.06, 95% CI: 0.88, 7.24), but there was little evidence of a difference between drug groups in the self (β = 1.95, −2.25, 6.16) or stranger conditions (β = 1.63, −2.46, 5.72).

**Fig. 3:**
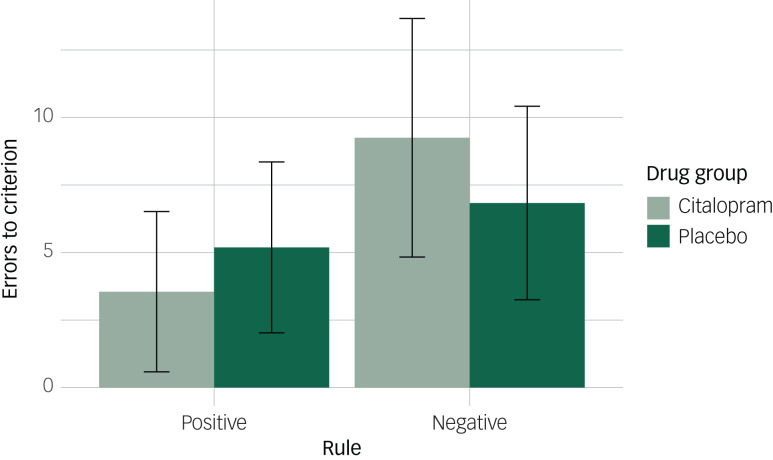
Mean errors to criterion in the friend condition according to drug group and rule. Error bars represent standard deviations.

To further explore the effect of drug group in the friend condition, we conducted additional exploratory analyses examining the effects of rule and drug group on errors to criterion for each referential condition separately. For the friend condition, an interaction between drug group and rule was observed (β = −4.06, 95% CI: −7.14, −0.98). Participants in the citalopram group on average made 3.55 (s.d. 2.97) errors before learning the ‘like’ rule and 9.25 (s.d. 4.42) errors before learning the ‘dislike’ rule. A smaller difference was observed between rules for the placebo group, with participants on average making 5.19 (s.d. 3.16) errors before learning the ‘like’ rule and 6.38 (s.d. 3.58) errors before learning the ‘dislike’ rule (Fig. 3). The greater positive bias towards the friend, observed with bias scores, was therefore driven by both reduced learning of the friend being disliked and increased learning of the friend being liked. We found no evidence for an interaction between drug group and rule when learning about the self (β = −1.95, 95% CI: −5.73, 1.83) or the stranger (β = −1.63, 95% CI: −5.16, 1.91).

However, these results are exploratory and require further replication to determine the reliability of the effects.

#### Referential emotional categorisation and recall

Participants endorsed a greater number of positive words as descriptive (β = −11.70, 95% CI: −13.50, −9.90, *P* <0.001), for both the self and other. There was no evidence that this effect differed between drug groups (Supplementary Table 3).

When examining the number of correctly recalled words, we found that participants recalled more positive than negative words (β = −1.30, 95% CI: −2.37, −0.23, *P* = 0.019). There was weak evidence that this differed according to referential condition, with participants recalling fewer dislikeable words in the other versus self condition, although the confidence interval included the null (β = −1.30, 95% CI: −2.82, 0.22, *P* = 0.096). We found no evidence of an interaction between valence and drug group, or referential condition and drug group (Supplementary Table 3).

**Fig. 4:**
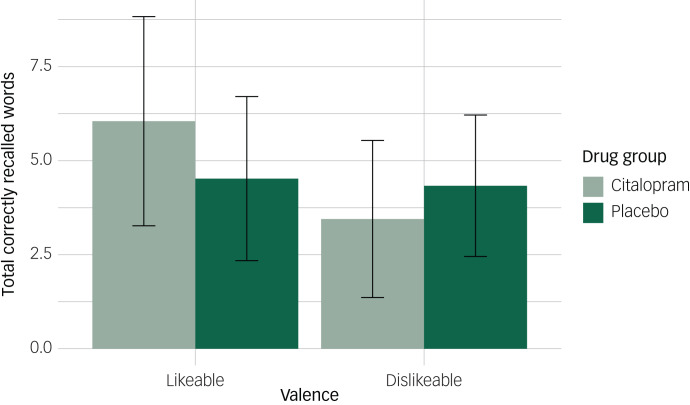
Mean number of correctly recalled words in the other condition according to drug group and valence. Error bars represent standard deviations.

There was weak evidence of an interaction between drug group, referential condition and valence (β = 1.92, 95% CI: −0.20, 4.04, *P* = 0.079), although the confidence interval included the null. To explore this effect further, we conducted additional exploratory analyses examining the interaction between drug group and valence for each referential condition separately. When recalling words about the self, participants recalled fewer dislikeable words (β = −1.30, 95% CI: −2.34, −0.27), but this did not vary by drug group (β = 0.49, 95% CI: −0.96, 1.94). Conversely, when recalling words about others, we observed an interaction between drug group and valence (β = 2.41, 95% CI: 0.89, 3.93). Participants in the citalopram group showed a positive bias towards others, recalling more likeable words and fewer dislikeable words, compared with the placebo group (Fig. 4). However, these findings are exploratory and require further replication.

The citalopram group had a higher proportion of participants that did not speak English as a first language (Table 1), which may have affected recall. Sensitivity analyses indicated that effects were consistent when this was taken into account. English as a first language was not associated with recall (β = −1.13, 95% CI: −2.48, 0.23, *P* = 0.112).

#### Go/no-go self-esteem

Ten participants were excluded from analyses for the go/no-go association self-esteem task as their responses indicated non-compliance according to *a priori* data exclusion criteria

Participants showed reduced discriminative accuracy for words relating to others versus self (β = −0.47, 95% CI: −0.84, −0.11, *P* = 0.012), and for negative versus positive words (although the confidence interval included the null; β = −0.36, 95% CI: −0.73, 0.00, *P* = 0.052). An interaction between referential condition and valence was observed (β = 0.65, 95% CI: 0.14, 1.16, *P* = 0.015). Participants showed higher discriminative accuracy for positive versus negative words in the self condition, but the opposite pattern in the other condition, suggesting a positive self-bias. However, there was no evidence that these effects varied by drug group (Supplementary Table 4).

Additional analyses suggested that these effects were driven by hits rather than false alarms (Supplementary Table 4).

When participants excluded according to *a priori* data exclusion criteria were included in the sensitivity analyses, the main and interactive effects of the referential condition and valence were further strengthened, but the lack of an effect of drug group remained.

#### Associative learning

A main effect of stimuli was observed for the self and emotion tasks, with participants showing greater accuracy and faster reaction times when matching shapes with the self and a happy face (Supplementary Table 5). For the reward task, there was no evidence of differences in accuracy according to level of reward, although participants were slightly slower to match shapes with the medium (£3) compared with the high (£9) level of reward. There was no evidence that performance on any of the associative learning tasks varied by drug group, either as a main effect or in interaction with stimuli (Supplementary Table 5).

### Blinding

A greater proportion of participants in the citalopram group (74%) believed that they had taken citalopram compared with the placebo group (14%), at a greater level of certainty (*t*(30.09) = 3.47, *P* = 0.002). Conversely, researchers administering the drug did not significantly differ in their group guesses and certainty according to drug group (Supplementary Table 6).

Failure of participant blinding was possibly attributable to the side-effects of citalopram, with the citalopram group only showing increases in nausea (*P* < 0.001) and dizziness (*P* = 0.012) over time. An increase in agitation (*P* = 0.011) and some evidence of an increase in headaches (*P* = 0.078) were also observed over time, but this varied little by group (*P* = 0.100 and *P* = 0.474, respectively). There was no evidence of changes for dry mouth or alertness in either group (Supplementary Table 7).

## Discussion

Our results tentatively support the theory that antidepressants increase positive affective biases and prosocial behaviours in healthy volunteers, in the absence of change in mood. Participants administered citalopram cooperated more in a prisoners’ dilemma game. There was also some evidence, based on exploratory analyses, that participants administered citalopram showed a greater positive bias when recalling words about others and when learning social evaluations about a friend.

In support of our pre-registered hypothesis, participants administered citalopram cooperated on a greater proportion of trials in a prisoners’ dilemma task. Our findings add to the literature indicating that serotonin is instrumental in modulating social behaviour. Previous research has indicated that temporary reduction of serotonin through tryptophan depletion reduces cooperative behaviours in a prisoners’ dilemma game.^40^ In line with our findings, increasing serotonin through administration of citalopram over a 2 week period has previously been found to increase cooperative communication and behaviour towards others.^18^ One week administration of an selective serotonin reuptake inhibitor has also been found to reduce self-reported hostile behaviours.^41^ Increases in prosocial behaviour resulting from antidepressant treatment may increase quality of social interactions, positively reinforcing engagement in interpersonal communications and increasing social support.

However, our findings of an increase in cooperative behaviours following a *single* dose of citalopram differs from those of previous research. In a previous study, a single dose of citalopram was not associated with greater cooperation in healthy volunteers.^42^ Disagreement between these findings may be attributable to variations in the prisoners’ dilemma task. In Tse and Bond's^42^ study, participants controlled the number of points allocated to the other player. In this study, allocation of points was dependent on both the participant's and the other player's decisions. Prosocial behaviours are believed to be motivated by the aim of eliciting reciprocal altruistic behaviours from others.^43^ In this study, participants may have therefore been more motivated to engage in cooperative actions, providing a more sensitive marker of change following serotonin modulation.

We also found some evidence that antidepressants increase prosocial affective biases towards familiar others. Participants administered citalopram showed better learning of friends being liked and reduced learning of friends being disliked by a computer persona. Affective recall was also altered. Participants administered citalopram recalled more likeable characteristics and fewer dislikeable characteristics of others. However, these were exploratory analyses and require further replication. One potential mechanism of antidepressants may involve blunting perceptions of negative characteristics and increasing sensitivity to positive characteristics in others, although this requires further exploration in clinical samples. Increasing positive perceptions of others through antidepressant treatment may increase engagement in social interactions, thereby addressing issues of social withdrawal and anhedonia associated with depression.

In contrast to our expectations, there was no evidence to support our hypothesis that the effects of antidepressants on affective processing were strongest for self-referential stimuli. This contrasts with previous research indicating a change in positive self-referential biases following antidepressant administration.^11,12^ However, in these studies, participants only encoded information in reference to the self. The specificity of this effect to self-related information is therefore unclear. In a study including both a self and friend condition, participants administered escitalopram endorsed fewer negative characteristics about themselves but also more positive characteristics about others.^44^ In this study, we found no evidence to suggest that citalopram selectively affected positive learning about the self. If anything, our exploratory analyses indicated that citalopram produced the largest group effects in the friend condition, with increased positive learning of social evaluations of friends following antidepressant treatment. However, our confirmatory test did not support a referential condition by drug group interaction.

Self-schemas are pervasive, dominate information processing and are resistant to disconfirmatory evidence.^1^ A single dose of an antidepressant may not be sufficient to address entrenched self-referential negative biases. Affective processing of information related to others may be more flexible and therefore more sensitive to change by acute administration of antidepressants. In support of this theory, pharmacological induction of anxiety was found to influence other-referential processing, whereas self-referential processing was preserved.^45^ We may therefore only see changes in self-referential affective biases with longer periods of antidepressant treatment. Alternatively, addressing negative self-schema may also require remediation of top-down, deliberative biases through treatments such as cognitive–behavioural therapy (CBT).^9^ Further longitudinal studies are required to examine changes in self-referential affective biases during long-term use of antidepressants, to assess their effectiveness in remediating negative self-schema.

We found no evidence that acute citalopram influenced inhibitory control, measured using an affective go/no-go association task, or simple associative learning of emotional, self and reward stimuli. Previous research has reported inconsistent findings regarding the relationship between depressive symptoms and performance on affective inhibitory control tasks.^46,47^ Similarly, we have previously found no association between simple associative learning and depression severity.^48^ Serotonin therefore appears to have little influence over these particular cognitive processes.

### Clinical implications

Variation in individual treatment response and delays in therapeutic action currently expose patients to considerable periods of potentially ineffective antidepressant treatment.^49^ Our findings suggest that changes in prosocial behaviours and, more tentatively, positive affective biases towards others may be sensitive markers of early changes in response to antidepressant treatment. Extension of our findings to clinical samples may offer a promising marker of treatment response that could allow clinicians to identify effective treatments for individuals experiencing depression at earlier timepoints.

Limited change in negative self-schema from antidepressant treatment, as we observed in this study, may provide an explanation for the relatively high relapse rates following treatment discontinuation.^50^ At present, it is unclear whether change in affective processing is sustained after antidepressant treatment has been discontinued. If increased positive affective biases do not translate to a sustained change in self-schema, depressive symptoms are likely to return following antidepressant discontinuation. Sustained changes in self-schema through treatments such as CBT may explain the substantially lower relapse rates compared with antidepressant treatment.^51^

### Limitations

This study had lower statistical power than planned, as recruitment was terminated prematurely owing to COVID-19. We were therefore powered to detect large effects and may have been underpowered to detect smaller effects such as the hypothesised interaction effects for social evaluation learning. To address this limitation, we have made all materials and data publicly available for further replication and extension of our work.

In addition, despite strict blinding procedures, there was evidence that blinding was not effective at a participant level. It is possible that our results may have been partially driven by response biases. However, mitigating this possibility, we predominantly used implicit affective tasks, where the main purpose of the tasks was not explicitly stated, and participants were blinded to the study hypotheses.

This study used a placebo drug as the comparator condition. However, high placebo response rates have been observed in antidepressant trials,^52^ suggesting that placebo expectations may influence similar cognitive processes. Future studies including a no-treatment-control arm condition or a placebo lead-in phase would allow more precise evaluation of the effects of citalopram on social cognition.

Finally, this study examined changes in social cognition and behaviour following antidepressant administration in healthy volunteers. The use of healthy volunteers offers the opportunity to study affective biases unconfounded by ‘cold’ cognitive biases.^53^ However, it limits our insight into the mechanisms of antidepressants in improving mood. It is also possible that our findings may not directly translate to clinically depressed patients. Further research would benefit from extending our findings to longer-term changes in affective processing in depressed individuals.

### Summary

Overall, our findings suggest that acute administration of citalopram in healthy volunteers is associated with increased prosocial behaviour towards others. In contrast to our expectations, we found little evidence that the effect of acute citalopram on affective processing was heightened for information related to the self. Exploratory analyses instead suggested increased positive affective biases towards others. Changes in affective processing and prosocial behaviours towards others may, at least partially, be a mechanism of antidepressant effect. Further research in clinical samples is required to examine this possibility.

## Data Availability

The data that support the findings of this study are openly available in the University of Bath Research Data Archive at https://doi.org/10.15125/BATH-00891, reference number 891.

## Supplementary material

For supplementary material accompanying this paper visit http://dx.doi.org/10.1192/bjo.2020.107. click here to view supplementary material
