# Supplementary material for: Effect of acute citalopram on self-referential emotional processing and social cognition in healthy volunteers
Source: BJPsych Open. 2020 Oct 19;6(6):e124. doi: 10.1192/bjo.2020.107 (PMC7576669; doi:10.1192/bjo.2020.107)
Supplement: Supplementary file 1 [file bjosup.zip › S2056472420001076sup001.docx]

**Supplementary Materials**

**Supplementary Methods**

*Detailed Description of Cognitive Tasks*

Study materials are available on Open Science Framework (<https://osf.io/nhjvs/>). Scripted text used by researchers to instruct participants for each cognitive task are available in the case report form, which is publicly available on Open Science Framework.

Prisoner’s Dilemma

Cooperative behaviours were measured using an iterated Prisoner’s Dilemma task (1,2). Participants were instructed that they had to choose to work with (cooperate) or against (defect) the computer to win points. On each trial, participants were shown a 2 x 2 matrix demonstrating the possible outcomes of players’ choices. Participants made their choice, before being shown the other’s choice and the resulting outcome. Points were structured so that the highest amount of points was won by defecting whilst the other chose to cooperate, followed in descending order by both players choosing to cooperate, both players choosing to defect, and choosing to cooperate whilst the other defected.

Participants completed two blocks of 26 trials, which differed according to social context. In one block the other player initially cooperated, whereas in a second block the other player initially defected. Two ‘other’ personas were used for each block. Order of social context and assignment of ‘other’ personae were counterbalanced. After the first trial, the other followed a ‘tit for tat’ strategy, mirroring the choice of the participant in the previous trial.

The proportion of cooperative choices and reaction times for cooperative choices were recorded.

Social Evaluation Learning

Learning of social evaluations was measured using a reinforcement learning task (3,4). Participants were told that they had to learn how much the computer liked themselves, a friend and a stranger. Separate blocks were completed for each referential condition. On each trial, participants were presented with positive-negative personality word pairs and were asked to select the word that represented the computer’s attitude. No time limit was imposed. Participants were given feedback on their selection (presented for 2000ms). For each referential condition, participants learnt varying levels of positive ‘like’ rules (60-80% of positive words correct) and negative ‘dislike’ rules (20-40% of positive words correct). Referential condition and rule order were randomised. 24 trials were completed per block.

Learning was assessed through the number of errors made before reaching the criterion of eight consecutive rule-congruent responses. This was averaged across each level of the positive ‘like’ and negative ‘dislike’ rules. Bias scores were calculated by subtracting errors to criterion made when learning the dislike rule from the like rule. A positive value indicates a negative bias, as fewer errors are made learning the dislike rule compared to the like rule. Conversely, a negative value indicates a positive bias, as fewer errors are made learning the like rule compared to the dislike rule.

Emotional Categorisation and Recall

Positive and negative words were selected from a dataset of personality trait descriptors rated for likability (5). Two lists of 20 positive and 20 negative words were created matched according to word length. At the beginning of the task participants were asked to enter the first name of a familiar other to allow personalised task instructions. Participants then encoded personality traits to the self or other by categorising whether presented positive and negative words described themselves/the other (“In this task we will ask you to indicate whether each word describes [self/other]”). Separate blocks were completed for each referential condition, with order and list assignment randomised. Participants were instructed to press a key to indicate if the word described the person (‘yes’) or did not describe the person (‘no’). ‘j’ and ‘k’ keys were used for input, with key assignment for each response randomised. Immediately following categorisation of personality traits participants were asked to recall as many of the presented characteristics as they could in two minutes, using the keyboard to enter their responses.

The total number of positive and negative words categorised as describing the self and other were recorded. The total number of positive and negative words correctly recalled were recorded according to referential condition.

Self-Esteem Go/No-Go Association

Inhibitory control when responding to affective words in relation to the self and others was measured using a go/no-go association task (6). This task was used to measure affective processing occurring in interaction with referential processing. Participants were instructed that they had to categorise words by pressing the spacebar. Participants categorised words relating to two referential conditions (self or others), and two emotional conditions (positive or negative characteristics). In each trial a word belonging to one of these categories was briefly displayed (600 ms) at the centre of the screen. Participants pressed the spacebar if the presented word belonged to a specified paired referential-emotion category (e.g. Self-Positive, Self-Negative, Other-Positive, Other-Negative). Four randomised blocks were completed relating to each referential-emotion combination, with 16 practice trials and 48 test trials per block. Response timeouts of 600 ms were applied.

Discriminative accuracy (d’) was calculated through applying z-score transformations and subtracting hit z-scores from false alarm z-scores for each referential-emotion combination. Z-scores were adjusted by adding or subtracting 0.005 if the values were 0 or 1 to remove extreme values.

Associative Learning

Simple associative learning of abstract shape pairings with self, reward and emotion was measured using three tasks (7,8). This was used to measure how self-reference, emotional valence, and reward independently influence simple associative learning. Previous work in healthy controls has found that associative learning is prioritised for the self, positive stimuli and high levels of reward (7,8). Nine practice trials and two blocks of 60 testing trials were completed per task.

In each task, participants were told that they had to match shapes with words or pictures. Shapes and stimuli varied according to each task; in the self-task, shapes were matched with the words ‘self’ ‘friend’ and ‘stranger’; in the emotion task, shapes were matched with happy, neutral and sad cartoon faces; and in the reward task, shapes were matched with high (£9), medium (£3) and low (£1) monetary rewards. Shape-stimuli pairings were randomly assigned.

Participants were presented with a combination of the stimuli-shape pairings and pressed the ‘n’ or the ‘m’ key to indicate whether the presented pairings matched with the previously learnt associations. In each trial a fixation point was displayed for 2000 ms, followed by a stimuli-shape pairing. Stimuli-shape pairings were presented for 100 ms for the self and reward associative tasks, and 150 ms for the emotion task due to the greater visual complexity of stimuli. Participants were asked to provide a response within 1100 ms and were then given feedback on their response for 500 ms. At the end of each block participants were informed of their accuracy (% correct).

For the reward task only, participants received a monetary reward based on the proportion of correct trials per category of reward stimuli.

*Detailed Description of Statistical Models for Cognitive Tasks*

Unless otherwise stated, analyses were pre-registered and confirmatory. For all models, subject was entered as a random effect to account for the repeated measures elements of the cognitive tasks. The citalopram group was used as the reference category in all analyses.

Prisoners’ Dilemma

Proportion of cooperative behaviours was the outcome, drug group, social context and the interaction between drug group and social context were predictors.

Social Evaluation Learning

Bias score was the outcome, and drug group, referential condition and the interaction between drug group and referential condition were predictors. Exploratory analyses were conducted examining the effect of drug group (the predictor) on bias score (the outcome) separately for each referential condition.

To understand whether effects on bias scores were driven by learning within a particular rule (e.g. better learning of ‘dislike’ or worse learning of ‘like’), we re-ran the models using errors to criterion as the outcome, and referential condition, rule, drug group and the interaction between these variables, as predictors. Exploratory analyses were conducted separately for each referential condition examining the effect of drug group, rule, and the interaction between drug group and rule on errors to criterion.

Referential Categorisation and Recall

The number of words categorised as descriptive (“yes”) was the outcome, and drug group, referential condition, valence, and the interaction between these were predictors. This model was repeated with the total number of words correctly recalled as the outcome. As the citalopram group had a higher proportion of participants that did not speak English as a first language, we conducted a sensitivity analysis repeating this model with a binary variable representing whether English was spoken as a first language (yes/no) included as an additional predictor variable.

Exploratory analyses were conducted separately for each referential condition, with total number of words correctly recalled as the outcome and valence, drug group and the interaction between drug group and valence as predictors.

Self-Esteem Go/No-Go Task

D’ was the outcome, and drug group, referential condition, valence, and the interaction between these were predictors. To assess whether effects on d’ may be driven by changes in hits or false alarms, these models were repeated separately with hits and false alarms as the outcome.

Associative Learning

Separate models were conducted for each task (self, emotion, reward) with accuracy (% correct) as the outcome. Stimuli, drug group, and the interaction between stimuli and drug group were predictors. These models were repeated with reaction times as the outcome.

**Supplementary Tables**

S1

*Follow-up Contrasts exploring the effect of Drug Group at each Timepoint on VAS ratings of Happiness and Sadness*

|  | **Estimate** | **SE** | **DF** | **t** | **p** |
| --- | --- | --- | --- | --- | --- |
| **Happiness** |  |  |  |  |  |
| Baseline | -8.22 | 3.94 | 57.8 | -2.09 | 0.041 |
| Post-Drug | -0.95 | 3.94 | 57.8 | -0.24 | 0.810 |
| Post-Testing | -2.26 | 3.94 | 57.8 | -0.57 | 0.568 |
| **Sadness** |  |  |  |  |  |
| Baseline | 8.86 | 3.75 | 66 | 2.36 | 0.021 |
| Post-Drug | 3.58 | 3.75 | 66 | 0.95 | 0.343 |
| Post-Testing | 4.57 | 3.75 | 66 | 1.22 | 0.227 |

Note: Citalopram used as the reference category for drug group.

S2

*Results from a mixed-effects regression analysis examining the effect of drug group, referential condition and rule on measures of learning in the Social Evaluation Learning Task*

|  | **β** | **95% CI** | **p** |
| --- | --- | --- | --- |
| **Bias Scores** |  |  |  |
| Intercept | -4.53 | -7.20, -1.85 | 0.001 |
| Drug group | 1.95 | -1.78, 5.69 | 0.308 |
| Referential Condition |  |  | 0.738 |
| *Self* | Reference |  |  |
| *Friend* | -1.18 | -3.91, 1.56 | 0.403 |
| *Stranger* | 0.35 | -2.39, 3.09 | 0.803 |
| Drug group * Referential Condition | |  | 0.387 |
| *Self* | Reference |  |  |
| *Friend* | 2.10 | -1.72, 5.93 | 0.284 |
| *Stranger* | -0.33 | -4.15, 3.50 | 0.868 |
| **Errors to Criterion** |  |  |  |
| Intercept | 4.13 | 2.37, 5.89 | < 0.001 |
| Drug group | 1.71 | -0.75, 4.17 | 0.175 |
| Referential Condition |  |  | 0.753 |
| *Self* | Reference |  |  |
| *Friend* | -0.58 | -2.89, 1.74 | 0.627 |
| *Stranger* | 1.10 | -1.21, 3.42 | 0.353 |
| Rule | 4.53 | 2.21, 6.84 | < 0.001 |
| Drug group * Referential Condition | |  | 0.585 |
| *Self* | Reference |  |  |
| *Friend* | -0.07 | -3.30, 3.17 | 0.967 |
| *Stranger* | -1.46 | -4.70, 1.78 | 0.379 |
| Drug group * Rule | -1.95 | -5.19, 1.28 | 0.238 |
| Referential Condition * Rule | |  | 0.845 |
| *Self* | Reference |  |  |
| *Friend* | 1.18 | -2.10, 4.45 | 0.483 |
| *Stranger* | -0.35 | -3.62, 2.93 | 0.834 |
| Drug group*Referential Condition*Rule | |  | 0.821 |
| *Self* | Reference |  |  |
| *Friend* | -2.10 | -6.68, 2.48 | 0.369 |
| *Stranger* | 0.33 | -4.25, 4.91 | 0.889 |

Note: Citalopram used as the reference category for drug group.

S3

*Results from a mixed-effects regression analysis examining the effect of drug group, referential condition and valence on total number of words categorised as descriptive and total number of words correctly recalled*

|  | **β** | **95% CI** | **p** |
| --- | --- | --- | --- |
| **Total Categorisations as Descriptive (“yes”)** |  |  |  |
| Intercept | 16.10 | 14.81, 17.40 | <0.001 |
| Drug Group | -0.20 | -2.01, 1.61 | 0.833 |
| Referential Condition | 0.35 | -1.45, 2.15 | 0.704 |
| Valence | -11.70 | -13.50, -9.90 | <0.001 |
| Drug Group * Referential Condition | 0.60 | -1.92, 3.12 | 0.640 |
| Drug Group * Valence | 0.89 | -1.63, 3.41 | 0.490 |
| Referential Condition * Valence | -1.25 | -3.80, 1.30 | 0.339 |
| Drug Group &* Referential Condition * Valence | 0.01 | -3.55, 3.58 | 0.995 |
| **Total Words Correctly Recalled** |  |  |  |
| Intercept | 6.10 | 5.06, 7.14 | <0.001 |
| Drug Group | -0.96 | -2.41, 0.50 | 0.200 |
| Referential Condition | -0.05 | -1.12, 1.02 | 0.927 |
| Valence | -1.30 | -2.37, -0.23 | 0.019 |
| Drug Group * Referential Condition | -0.57 | -2.07, 0.93 | 0.459 |
| Drug Group * Valence | 0.49 | -1.01, 1.99 | 0.523 |
| Referential Condition * Valence | -1.30 | -2.82, 0.22 | 0.096 |
| Drug Group &* Referential Condition * Valence | 1.92 | -0.20, 4.04 | 0.079 |

Note: Citalopram used as the reference category for drug group, self used as reference category for referential condition, positive (‘likeable’) used as reference category for valence.

S4

*Results from a mixed-effects regression analysis examining the effect of drug group, referential condition and valence on discriminative accuracy (D’), hits (%), and false alarms (%) in a Go/No-Go Association Self-Esteem Task*

|  | **β** | **95% CI** | **p** |
| --- | --- | --- | --- |
| **D’** |  |  |  |
| Intercept | 1.31 | 1.03, 1.60 | < 0.001 |
| Drug Group | 0.20 | -0.20, 0.59 | 0.339 |
| Referential Condition | -0.47 | -0.84, -0.11 | 0.012 |
| Valence | -0.36 | -0.73, 0.00 | 0.052 |
| Drug Group * Referential Condition | -0.24 | -0.74, 0.27 | 0.362 |
| Drug Group * Valence | -0.41 | -0.92, 0.09 | 0.112 |
| Referential Condition * Valence | 0.65 | 0.14, 1.16 | 0.015 |
| Drug Group &* Referential Condition * Valence | 0.45 | -0.27, 1.16 | 0.223 |
| **Hits (%)** |  |  |  |
| Intercept | 72.22 | 65.66, 78.79 | < 0.001 |
| Drug Group | 8.77 | -0.37, 17.90 | 0.064 |
| Referential Condition | -10.00 | -17.09, -2.91 | 0.007 |
| Valence | -8.33 | -15.42, -1.24 | 0.024 |
| Drug Group * Referential Condition | -4.58 | -14.45, 5.29 | 0.365 |
| Drug Group * Valence | -7.29 | -17.16, 2.58 | 0.151 |
| Referential Condition * Valence | 16.94 | 6.92, 26.97 | 0.001 |
| Drug Group * Referential Condition * Valence | 5.71 | -8.25, 19.67 | 0.425 |
| **False Alarms (%)** |  |  |  |
| Intercept | 28.61 | 21.69, 35.54 | < 0.001 |
| Drug Group | -0.49 | -10.12, 9.15 | 0.922 |
| Referential Condition | 7.22 | -0.31, 14.75 | 0.063 |
| Valence | 1.39 | -6.14, 8.92 | 0.719 |
| Drug Group * Referential Condition | 1.11 | -9.37, 11.59 | 0.836 |
| Drug Group * Valence | 9.03 | -1.45, 19.51 | 0.095 |
| Referential Condition * Valence | -6.94 | -17.59, 3.70 | 0.204 |
| Drug Group &* Referential Condition * Valence | -5.56 | -20.37, 9.26 | 0.464 |

Note: Citalopram used as the reference category for drug group, self used as reference category for referential condition, positive used as reference category for valence.

S5

*Results from a mixed-effects regression analysis examining the effect of drug group and stimuli on accuracy (%) and reaction times (ms) in associative learnings tasks of self, emotion and reward*

|  | **Accuracy (%)** | | | **Reaction Times (ms)** | | |
| --- | --- | --- | --- | --- | --- | --- |
|  | **β** | **95% CI** | **p** | **β** | **95% CI** | **p** |
| **Self** |  |  |  |  |  |  |
| Intercept | 89.96 | 85.03, 94.89 | < 0.001 | 696.36 | 664.61, 728.10 | < 0.001 |
| Drug Group | 1.30 | -5.59, 8.18 | 0.713 | -11.51 | -55.87, 32.85 | 0.613 |
| Stimuli |  |  | < 0.001 |  |  | < 0.001 |
| Self | Reference |  |  |  |  |  |
| Friend | -5.59 | -10.36, -0.82 | 0.024 | 28.12 | 10.71, 45.53 | 0.002 |
| Stranger | -10.59 | -15.36, -5.82 | < 0.001 | 39.60 | 22.19, 57.01 | < 0.001 |
| Drug Group * Stimuli |  |  | 0.503 |  |  | 0.812 |
| Self | Reference |  |  |  |  |  |
| Friend | -3.76 | -10.43, 2.90 | 0.272 | 7.79 | -16.54, 32.12 | 0.532 |
| Stranger | -0.98 | -7.65, 5.69 | 0.774 | 3.16 | -21.17, 27.49 | 0.800 |
| **Emotion** |  |  |  |  |  |  |
| Intercept | 80.81 | 73.81, 87.81 | < 0.001 | 731.27 | 687.21, 775.34 | < 0.001 |
| Drug Group | 0.08 | -9.71, 9.86 | 0.988 | -8.59 | -70.16, 52.98 | 0.786 |
| Stimuli |  |  | < 0.001 |  |  | < 0.001 |
| Happy | Reference |  |  |  |  |  |
| Neutral | -12.61 | -18.59, -6.64 | < 0.001 | 48.11 | 25.63, 70.58 | < 0.001 |
| Sad | -12.45 | -18.43, -6.47 | < 0.001 | 45.79 | 23.32, 68.27 | < 0.001 |
| Drug Group * Stimuli |  |  | 0.716 |  |  | 0.463 |
| Happy | Reference |  |  |  |  |  |
| Neutral | -2.71 | -11.07, 5.64 | 0.526 | -19.45 | -50.85, 11.95 | 0.228 |
| Sad | -3.14 | -11.50, 5.21 | 0.463 | -10.45 | -41.85, 20.95 | 0.516 |
| **Reward** |  |  |  |  |  |  |
| Intercept | 81.41 | 75.32, 87.51 | <0.001 | 707.51 | 670.14, 744.88 | < 0.001 |
| Drug Group | -4.01 | -12.53, 4.51 | 0.359 | -23.29 | -75.50, 28.93 | 0.386 |
| Stimuli |  |  | 0.080 |  |  | 0.008 |
| High (£9) | Reference |  |  |  |  |  |
| Medium (£3) | -5.15 | -11.35, 1.06 | 0.108 | 25.44 | 1.30, 49.59 | 0.042 |
| Low (£1) | -4.01 | -10.22, 2.20 | 0.209 | 10.20 | -13.95, 34.34 | 0.410 |
| Drug Group * Stimuli |  |  | 0.372 |  |  | 0.750 |
| High (£9) | Reference |  |  |  |  |  |
| Medium (£3) | -0.56 | -9.23, 8.11 | 0.900 | 12.52 | -21.21, 46.25 | 0.469 |
| Low (£1) | 4.98 | -3.69, 13.66 | 0.264 | 8.31 | -25.42, 42.04 | 0.630 |

Note: Citalopram used as the reference category for drug group.

S6

*Group assignment guesses and certainty ratings made by participants and researchers according to drug group*

|  | Citalopram (N = 19)^a^ | Placebo (N = 21) | p |
| --- | --- | --- | --- |
| **Participant** |  |  |  |
| Group Guess, N (%) |  |  | X^2^(1) = 12.07, p < .001 |
| Citalopram | 14 (74) | 3 (14) |  |
| Placebo | 5 (26) | 18 (86) |  |
| Certainty, M (SD) |  |  |  |
| Citalopram | 53.74 (31.49) | 24.38 (20.14) | t(30.09)=3.47, p = 0.002 |
| Placebo | 30.74 (29.63) | 55.14 (22.99) | t(33.89)=-2.89, p = 0.007 |
| **Researcher** |  |  |  |
| Group Guess, N (%) |  |  | X^2^(1) = 0.06, p = 0.806 |
| Citalopram | 8 (42) | 7 (33) |  |
| Placebo | 11 (58) | 14 (66) |  |
| Certainty, M (SD) |  |  |  |
| Citalopram | 29.32 (27.62) | 28.86 (19.35) | t(31.88) = 0.06, p = 0.952 |
| Placebo | 22.79 (23.65) | 33.24 (22.73) | t(37.25) = -1.42, p = 0.164 |

^a^ Data for one participant unavailable due to a technical error, total N = 20

S7

*Results from a mixed-effects regression analysis examining the effect of drug group and timepoint on self-reported side effects*

|  | **β** | **95% CI** | **p** |
| --- | --- | --- | --- |
| **Nausea** |  |  |  |
| Intercept | 1.00 | 0.80, 1.20 | < 0.001 |
| Drug Group | 0.00 | -0.27, 0.27 | 1.000 |
| Timepoint |  |  | < 0.001 |
| Baseline | Reference |  |  |
| Post-Drug | 0.68 | 0.45, 0.92 | < 0.001 |
| Post-Testing | 0.47 | 0.24, 0.70 | < 0.001 |
| Drug Group * Timepoint | |  | < 0.001 |
| Baseline | Reference |  |  |
| Post-Drug | -0.64 | -0.95, 0.32 | < 0.001 |
| Post-Testing | -0.47 | -0.79, -0.16 | 0.005 |
| **Dizziness** |  |  |  |
| Intercept | 1.05 | 0.86, 1.24 | < 0.001 |
| Drug Group | -0.01 | -0.27, 0.26 | 0.970 |
| Timepoint |  |  | < 0.001 |
| Baseline | Reference |  |  |
| Post-Drug | 0.53 | 0.31, 0.74 | < 0.001 |
| Post-Testing | 0.32 | 0.10, 0.53 | 0.005 |
| Drug Group * Timepoint | |  | 0.012 |
| Baseline | Reference |  |  |
| Post-Drug | -0.43 | -0.73, -0.14 | 0.005 |
| Post-Testing | -0.32 | -0.61, - 0.02 | 0.039 |
| **Dry Mouth** |  |  |  |
| Intercept | 1.32 | 1.10, 1.53 | < 0.001 |
| Drug Group | -0.17 | -0.47, 0.13 | 0.262 |
| Timepoint |  |  | 0.410 |
| Baseline | Reference |  |  |
| Post-Drug | -0.11 | -0.34, 0.13 | 0.377 |
| Post-Testing | -0.21 | -0.44, 0.02 | 0.080 |
| Drug Group * Timepoint | |  | 0.522 |
| Baseline | Reference |  |  |
| Post-Drug | 0.15 | -0.17, 0.47 | 0.353 |
| Post-Testing | 0.16 | -0.16, 0.48 | 0.323 |
| **Headache** |  |  |  |
| Intercept | 1.00 | 0.84, 1.16 | < 0.001 |
| Drug Group | 0.05 | -0.18, 0.27 | 0.679 |
| Timepoint |  |  | 0.078 |
| Baseline | Reference |  |  |
| Post-Drug | 0.26 | 0.06, 0.47 | 0.013 |
| Post-Testing | 0.21 | 0.01, 0.41 | 0.046 |
| Drug Group * Timepoint | |  | 0.474 |
| Baseline | Reference |  |  |
| Post-Drug | -0.17 | -0.45, 0.11 | 0.246 |
| Post-Testing | -0.12 | -0.40, 0.17 | 0.424 |
| **Alertness** |  |  |  |
| Intercept | 2.26 | 1.85, 2.68 | < 0.001 |
| Drug Group | 0.12 | -0.45, 0.69 | 0.687 |
| Timepoint |  |  | 0.467 |
| Baseline | Reference |  |  |
| Post-Drug | -0.21 | -0.58, 0.16 | 0.266 |
| Post-Testing | 0.00 | -0.37, 0.37 | 1.000 |
| Drug Group * Timepoint | |  | 0.928 |
| Baseline | Reference |  |  |
| Post-Drug | -0.03 | -0.54, 0.48 | 0.916 |
| Post-Testing | -0.10 | -0.60, 0.41 | 0.715 |
| **Agitation** |  |  |  |
| Intercept | 1.00 | 0.84, 1.16 | < 0.001 |
| Drug Group | 0.00 | -0.23, 0.23 | 1.000 |
| Timepoint |  |  | 0.011 |
| Baseline | Reference |  |  |
| Post-Drug | 0.21 | 0.01, 0.41 | 0.040 |
| Post-Testing | 0.11 | -0.09, 0.30 | 0.300 |
| Drug Group * Timepoint | |  | 0.100 |
| Baseline | Reference |  |  |
| Post-Drug | -0.12 | -0.39, 0.16 | 0.411 |
| Post-Testing | 0.18 | -0.09, 0.45 | 0.199 |

Note: Citalopram used as the reference category for drug group.

**Supplementary Figures**

F1

Mean bias scores according to referential condition and drug group in the Social Evaluation Learning task. Greater bias scores indicate relatively better learning of the negative versus positive rule. Error bars represent standard deviations.

**References**

1. Wood RM, Rilling JK, Sanfey AG, Bhagwagar Z, Rogers RD. Effects of tryptophan depletion on the performance of an iterated Prisoner’s Dilemma game in healthy adults. Neuropsychopharmacology. 2006;31(5):1075–84.

2. Rilling JK, Gutman DA, Zeh TR, Pagnoni G, Berns GS, Kilts CD. A neural basis for social cooperation. Neuron. 2002;35(2):395–405.

3. Button KS, Browning M, Munafò MR, Lewis G. Social inference and social anxiety: Evidence of a fear-congruent self-referential learning bias. J Behav Ther Exp Psychiatry. 2012;43(4):1082–7.

4. Button KS, Kounali D, Stapinski L, Rapee RM, Lewis G, Munafò MR. Fear of negative evaluation biases social evaluation inference: Evidence from a probabilistic learning task. PLoS One. 2015;10(4):e0119456.

5. Anderson NH. Likeableness Ratings of 555 Personality-Trait Words. J Pers Soc Psychol. 1968;9(3):272.

6. Gregg AP, Sedikides C. Narcissistic fragility: Rethinking its links to explicit and implicit self-esteem. Self Identity. 2010;9(2):142–61.

7. Sui J, Ohrling E, Humphreys GW. Negative mood disrupts self- and reward-biases in perceptual matching. Q J Exp Psychol. 2016;69(7):1438–48.

8. Stolte M, Humphreys G, Yankouskaya A, Sui J. Dissociating biases towards the self and positive emotion. Q J Exp Psychol. 2017;70(6):1011–22.
